# Network effects of traumatic brain injury: from infra slow to high frequency oscillations and seizures

**DOI:** 10.1007/s10827-025-00895-5

**Published:** 2025-02-28

**Authors:** Brianna Marsh, Sylvain Chauvette, Mingxiong Huang, Igor Timofeev, Maxim Bazhenov

**Affiliations:** 1 Neuroscience Graduate Program, The University of California San Diego, 9500 Gilman Dr, La Jolla, San Diego, CA 92093 USA; 2 Department of Psychiatry and Neuroscience, Université Laval, 2325 Rue de l’Université, Québec, QC G1V 0A6 Canada; 3 Department of Radiology, The University of California San Diego, 9500 Gilman Dr, La Jolla, San Diego, CA 92093 USA; 4 Department of Medicine, The University of California San Diego, 9500 Gilman Dr, La Jolla, San Diego, CA 92093 USA

**Keywords:** TBI, Oscillations, Delta, Gamma

## Abstract

Traumatic brain injury (TBI) can have a multitude of effects on neural functioning. In extreme cases, TBI can lead to seizures both immediately following the injury as well as persistent epilepsy over years to a lifetime. However, mechanisms of neural dysfunctioning after TBI remain poorly understood. To address these questions, we analyzed human and animal data and we developed a biophysical network model implementing effects of ion concentration dynamics and homeostatic synaptic plasticity to test effects of TBI on the brain network dynamics. We focus on three primary phenomena that have been reported *in vivo* after TBI: an increase in infra slow oscillations (<0.1 Hz), increase in Delta power (1 - 4 Hz), and the emergence of broadband Gamma bursts (30 - 100 Hz). Using computational network model, we show that the infra slow oscillations can be directly attributed to extracellular potassium dynamics, while the increase in Delta power and occurrence of Gamma bursts are related to the increase in strength of synaptic weights from homeostatic synaptic scaling triggered by trauma. We also show that the buildup of Gamma bursts in the injured region can lead to seizure-like events that propagate across the entire network; seizures can then be initiated in previously healthy regions. This study brings greater understanding of the network effects of TBI and how they can lead to epileptic activity. This lays the foundation to begin investigating how injured networks can be healed and seizures prevented.

**Supplementary Information:**

The online version contains supplementary material available at 10.1007/s10827-025-00895-5.

## Introduction

Traumatic Brain Injury (TBI) can come in many different forms and from many different sources, such as rotational shearing, blunt force or incision, or internal bleeding (Saatman et al., 2008). In common, however, is the ability to disrupt normal neural functioning and activity. Some TBI’s can cause abnormal synchrony that escalates to seizures both immediately following the trauma and after a long time delay - months or even years later (Chauvette et al., 2016; Avramescu and Timofeev, 2008; Holden et al., 2021; Dinner, 1993). While treatments exist for epilepsy, up to 30% of patients are drug resistant in the long term (Łukawski et al., 2018). One approach to prevent seizures following TBI is to administer antiepileptic drugs (AEDs) that inhibit neural activity (Chartrain et al., 2017; Topolnik et al., 2003a); however, the efficacy of this treatment has been called into question (Wat et al., 2019; Temkin et al., 1990). Due to this, novel approaches of intervention following injury that address abnormal network behavior and the underlying pathology are needed. To address these problems, we first need to characterize the mechanisms of abnormal network behavior after injury.

Following TBI, marked changes in oscillatory activity have been well characterized (Franke et al., 2016; Guerriero et al., 2022; Huang et al., 2019; Lewine et al., 1999; Modarres et al., 2017). In particular, Kaltiainen et al. (2018) noted an increase in slow oscillations and theta in the same region as TBI lesions. Even more dramatically, Lewine et al. (2007) showed that subjects with post concussive symptoms lasting over a year may still exhibit abnormal activity. Increased slow oscillations have further been associated with working memory impairments in post-traumatic amnesia (Mallas et al., 2022). Furthermore, hyperactivity in the Gamma range (30-80 Hz) has been measured in patients with post-concussion syndrome and in task-evoked MEG during working memory tasks (Huang et al., 2019; Culic et al., 2005; Bailey et al., 2017; Huang et al., 2020). Finally, many studies have reported an increase in Delta range activities following brain lesions (Gloor et al., 1977; Gosselin et al., 2009; Safar et al., 2021; Sandsmark et al., 2017; Thomasy et al., 2016; Sarasso et al., 2020). In a study of high school football players, a single concussion during a season of play can increase Delta activity on an individual basis (Davenport et al., 2022). These studies show that abnormal oscillatory activity following TBI can be a pervasive and persistent outcome; we here aim to explore the mechanisms of these abnormal oscillations across different frequency bands.

Homeostatic Synaptic Plasticity (HSP) was identified as the generic regulatory mechanism to maintain network excitability by alternating balance of excitation and inhibition in brain networks (Burrone and Murthy, 2003; Pozo and Goda, 2010; Turrigiano, 2008). The sudden lack of synaptic input to the injured neurons following TBI triggers HSP that shifts the balance of remaining synaptic connections toward excitation in an attempt to regain a normal level of excitability (Avramescu and Timofeev, 2008; Timofeev et al., 2013; Topolnik et al., 2003a). It has been proposed that in the extreme case of traumatic brain injury, HSP may fail to compensate properly for the lost inputs, inducing over-excitation and driving seizures (Avramescu and Timofeev, 2008; Timofeev et al., 2013; Topolnik et al., 2003a; Fröhlich et al., 2008b; González et al., 2019; Houweling et al., 2005; Volman et al., 2013, 2011b, a; Nita et al., 2007). We have further shown that a complex pattern of seizure-like events after TBI depends on the ion concentration dynamics and increase of network excitability can trigger a positive feedback loop leading to the elevation of extracellular potassium concentration and seizures (Bazhenov et al., 2008, 2004, 2002; Frohlich et al., 2010; Fröhlich et al., 2006; Fröhlich and Bazhenov, 2006). HSP and extracellular potassium concentration are both heavily dependent on (and regulators of) cellular firing rate - when traumatic injury occurs and firing rate drops dramatically, both may become disrupted and have unanticipated effects. We thus propose that compensatory HSP and elevated extracellular potassium may further be key mechanisms in generating abnormal oscillatory activity (Gamma, Delta, etc) that is seen following TBI regardless of whether or not seizures will later occur.

In this new study, we integrate and extend our previous results to test how TBI-related disruption of ion concentration dynamics and synaptic homeostasis may contribute to the complex brain dynamics changes observed after TBI, particularly the increase in infra-slow oscillations, Delta waves, and Gamma bursts, all the way to seizure activity. We first examine experimental evidence from studies in cats and humans. These data sources independently show consistent upregulation of both the Delta and Gamma frequency range activities after TBI. We then developed a model of traumatic brain injury that includes ion concentration dynamics and HSP to test the hypothesis that the interplay of HSP-dependent strengthening of synaptic connections after TBI and oscillations of the extracellular $K^+$ concentration may explain the pathological increase of brain network synchronization as observed after TBI *in vivo*. In particular, we focus on the infra-slow (<0.1 Hz), Delta (1 - 4 Hz), and Gamma (30 - 100 Hz) frequency bands. We found that synaptic deafferentation of the injury zone, simulating effect of lesion, leads to spontaneous gamma bursts resembling high-frequency oscillation (HFO) interictal events (Houweling et al., 2005; Volman et al., 2013), grouped in the Delta frequency band and nested by elevated infra-slow oscillations. For severe injuries concentrated within a smaller brain region, these Gamma bursts triggered spontaneous spike-and-wave seizures. Our study characterized the spatiotemporal dynamics of pre-seizure interictal events and revealed the underlying mechanisms of their generation in the traumatized brain.

**Fig. 1:**
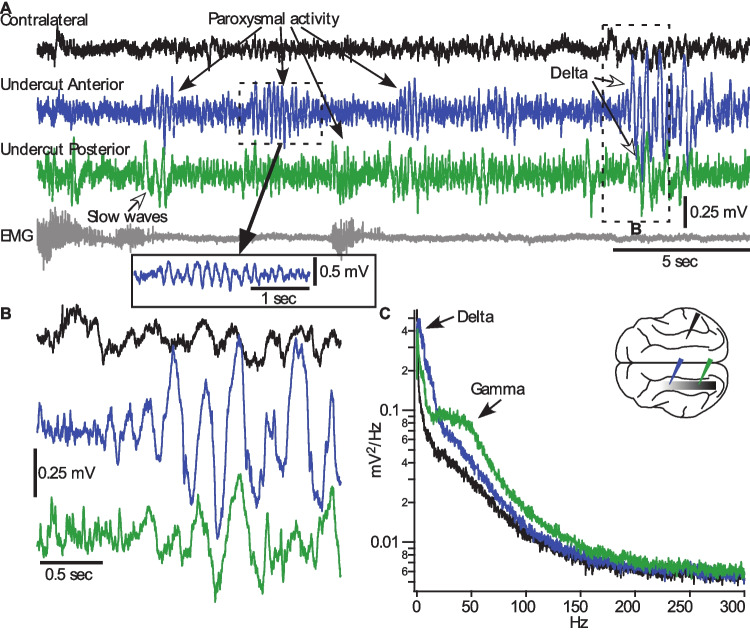
Increased delta and broad band gamma activities in the undercut cortex of the awake cat. (A) A segment of LFP activities recorded from a freely moving cat with cortical undercut located in the left suprasylvian cortex (see figurine in C). Green, posterior, largely deafferented cortex; blue, anterior, relatively intact cortex and black, intact contralateral cortex. Grey trace - electromyogram (EMG). Note that the undercut cortex displays occasional delta waves with a leading frequency of 2-4 Hz and rhythmic paroxysmal events with a frequency of 6-8 Hz. One of the paroxysmal events is expanded as indicated by an arrow. (B) An expanded segment from A, showing large amplitude delta waves in the undercut cortex during wake. (C) Power spectral analysis (Fast Fourier Transformation) of a 10 min segment of quiet wakefulness. Color code is the same as in A

## Results

### Experimental data

#### *In vivo* recordings from cats

Using wireless transmitting amplifiers, we recorded LFP activities from several cortical areas in freely behaving cats that had undergone an undercut that serves as a model of TBI we previously proposed (Timofeev et al., 2013; Nita et al., 2007; Kuśmierczak et al., 2015; Topolnik et al., 2003b; Nita et al., 2006). The undercut is a surgical lesion that was done in the left suprasylvian gyrus to replicate features commonly observed in human TBI. Because the cutting knife was inserted from posterior to anterior direction, the deafferentation in the posterior part was much bigger than in the anterior part (Akoglu, 2018). Our previous studies demonstrated that (a) 70% of fully adult cats had acute seizures occurring within the first 8-24 hours from the undercut (Topolnik et al., 2003b) followed by a period without seizures lasting 3-6 weeks after which the cats became epileptic with 1-3 spontaneous seizures per day (Nita et al., 2007; b) seizures started either in the anterior, relatively intact part of the undercut or in areas surrounding the undercut cortex and show consistent overall increase in slow-wave activity around undercut cortex (Nita et al., 2007, 2006; Timofeev et al., 2013). In this study we analyzed periods specifically preceding the onset of seizures. Here we report that undercut cortex, in particular the anterior part (relatively intact area) displayed (a) increased activities in the slow/delta frequency range and (b) increased power of broad band gamma activities (Fig. 1). Such an increase in delta activity was constantly observed in quiet wakefulness, and occasionally spread to the entire hemisphere in which only one gyrus was undercut (Timofeev et al., 2013). Delta waves in the undercut cortex were infrequent in active wakefulness and were virtually absent during REM sleep. This observation is congruent with a recent human study showing sleep-like TMS responses in awake patients with brain trauma in perilesional, but not contralateral cortex (Sarasso et al., 2020).

**Fig. 2:**
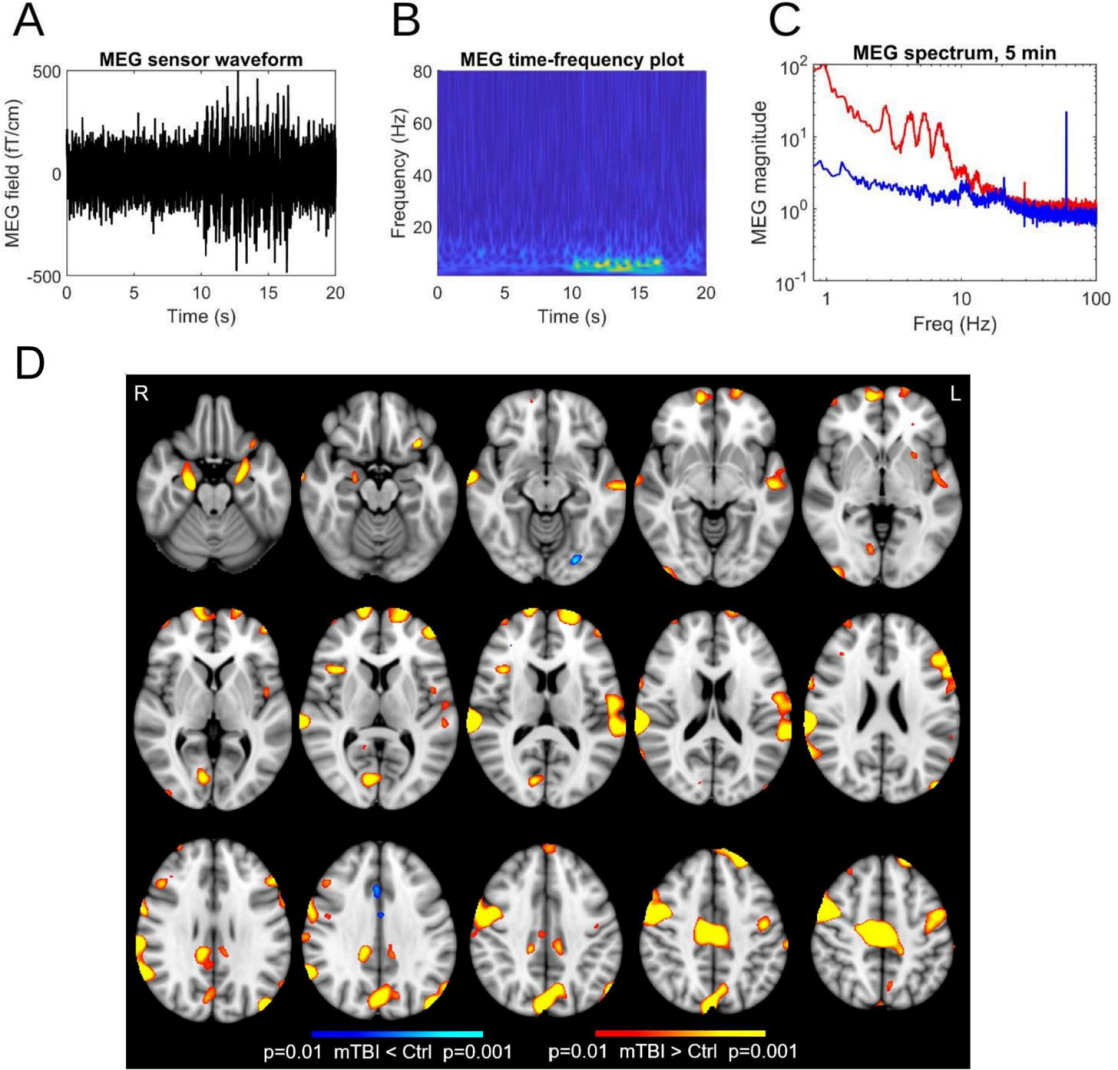
Increased delta and gamma power in human subjects with blast TBI. (A) rsMEG sensor waveform showing slow waves from one TBI patient. (B) Time-frequency plot of the slow waves. (C) Spectra plots of 5-min rsMEG recordings from a TBI (red) and a control (blue) subject. There are significant increases in both the delta and gamma bands (p < 0.01). Spike is 60 Hz powerline artifact. (D) Abnormal increases (regions with red-yellow color) of rsMEG delta-wave (1-4 Hz) activity in 26 blast TBI veterans, compared with 19 healthy control veterans. Abnormal increases were seen in bilateral hippocampi, frontal poles, dorsolateral prefrontal cortex, superior and middle temporal gyri, primary motor cortex, supplementary motor area, precuneous, and left orbitofrontal cortex

#### Human recordings

We further analyzed data from 26 veterans with blast-related symptomatic TBI and 19 healthy control veterans with similar deployment experiences. MEG sensor waveforms from a representative patient with TBI showed an increased slow-wave “burst” (Fig. 2A); the time-frequency plot shows the slow-wave activity was mainly in the delta band and extended to the theta band (Fig. 2B). The MEG spectrum plot during a 5-min recording session from a TBI and a healthy control subject (Fig. 2C) shows the increase of slow-wave activity mainly in delta (p < 0.01) but extending to theta bandwidths. The multiple peaks between 1-7 Hz (red curve) in Fig. 2C show abnormal delta (1-4 Hz) that has extended to theta-band (4-7 Hz) activity in the mTBI subject, and may be due to more than one generator of abnormal low-frequency activity. These findings showed that blast-related TBI resulted in abnormal increases of delta-band rsMEG activation. The TBI patient also showed a statistically significant increase in the gamma band (excluding the 60 Hz artifact) when compared with the healthy control participant (p < 0.01). While the increase in Gamma is not as clearly apparent, differences in Gamma are more difficult to detect - higher frequency signals lose power quickly when large regions are averaged over, as is inherent to MEG data (as opposed to invasive cortical electrodes as is used in the cats, or the single cell resolution of a computational models). For this reason, it is expected that increases in Gamma will be less obvious when working with human MEG data. Figure 2D shows the results from the group analysis of voxel-wise ANOVA that compared rsMEG activity in the delta band for all 26 veterans with blast-related symptomatic TBI and all 19 healthy control veterans with similar deployment experiences. While none of the subjects (mTBI or control) had visible lesions in the structural MRI, the mTBI subjects showed more frequent abnormal MEG signals from bilateral hippocampi, frontal poles, superior temporal gyri, primary motor cortices, mid-line supplemental motor area and cuneus and precuneus gyri.

As a whole, these two independent studies showed consistent pathology as a result of varied forms of TBI across species and measurement techniques - in particular, increased Delta and broadband Gamma power. We next turned to computational modeling to further delve into possible mechanistic explanations for these observed phenomena.

**Fig. 3:**
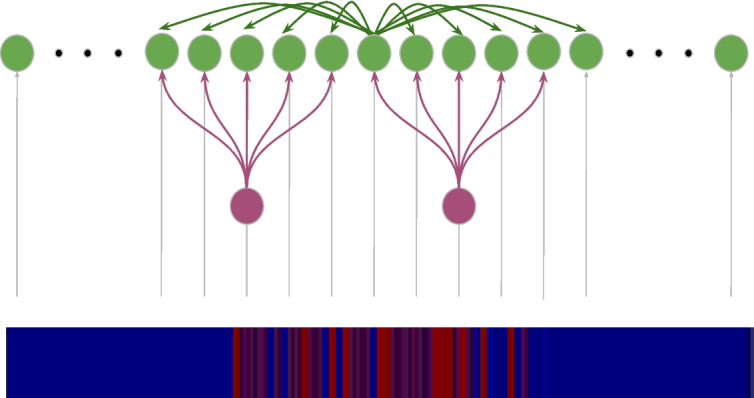
(Top) Linear model diagram, depicting each pyramidal cell (green) has a connection radius of 5 to other pyramidal cells (10 total connections). Each inhibitory cell (purple) connects to 5 total pyramidal cells. There are 200 pyramidal and 40 inhibitory neurons in total. (Bottom) Trauma pattern diagram, depicting location of the 40 injured neurons (red lines) across all 200 linear pyramidal neurons

### Steady random activity in control model

The model consists of 200 pyramidal neurons and 40 inhibitory neurons arranged as a linear array; pyramidal neurons have a connection radius of 5, while each inhibitory neuron synapses onto 5 excitatory neurons (Fig. 3). The model neurons are conductance-based two compartment models simulating dynamics of regular spiking pyramidal neurons and fast-spiking inhibitory interneurons. Ion concentration dynamics, including $Na^+$/$K^+$ exchanger, KCC2 pump, and glial buffering of extracellular potassium, are simulated to calculate reversal potentials for all ionic currents (see Methods). Local Homeostatic Synaptic Plasticity (HSP) works to maintain a steady firing rate by adjusting the strength of connections between pyramidal neurons (see Methods). All neurons further receive steady Poisson (external) input to simulate long-range connections from other brain areas.

**Fig. 4:**
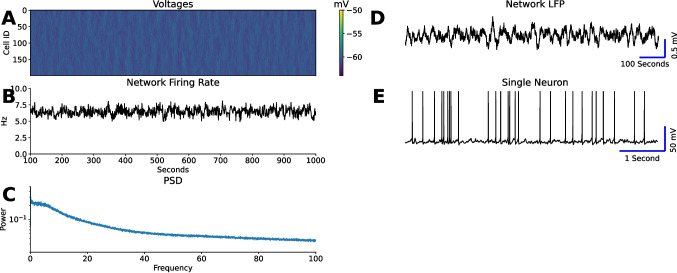
Dynamics of a healthy control network with steady, homogenous activity throughout the network. (A-B) Raster plot of cell voltages (A) and mean network firing rate (B) are shown for 1000 sec simulation. (C-D) The power spectral density (PSD) reveals a characteristic 1/f phenomenon (C), while the local field potential (LFP) shows very slow (<0.1 Hz) oscillations (D). (E) A characteristic single neuron voltage trace shows spontaneous tonic firing activity

Traumatic Brain Injury (TBI) is implemented as a 50% reduction of external input to cortical neurons. We previously proposed this model (González et al., 2015; Houweling et al., 2005; Volman et al., 2013, 2011b, a) to simulate the effect of cortical undercut, as reported here in animal data. In most of the simulations (except Fig. 10), the effect of TBI is applied to a random 40 of the 80 most central neurons to simulate a diffuse injury pattern (Fig. 3). These central 80 neurons will be referred to below as the Injury Zone, while the 60 uninjured neurons on either side will be considered the Healthy Zones. All model simulations discussed in this paper are replicated 10 times with different random seeds.

The model dynamics in a healthy, uninjured state is characterized in Fig. 4. The model successfully utilized local HSP mechanisms to maintain a steady moderate firing rate which was homogenous throughout the network. The power spectral density revealed a strong 1/f phenomenon, a hallmark of biological neural data (Buzsáki, 2006). Single neuron voltage traces showed steady, regular firing while the network local field potential (LFP) revealed very slow low-amplitude oscillations pervasive throughout the simulation. We have previously shown that these infra-slow oscillations are strongly tied to the extracellular potassium fluctuations (Krishnan et al., 2018).

**Fig. 5:**
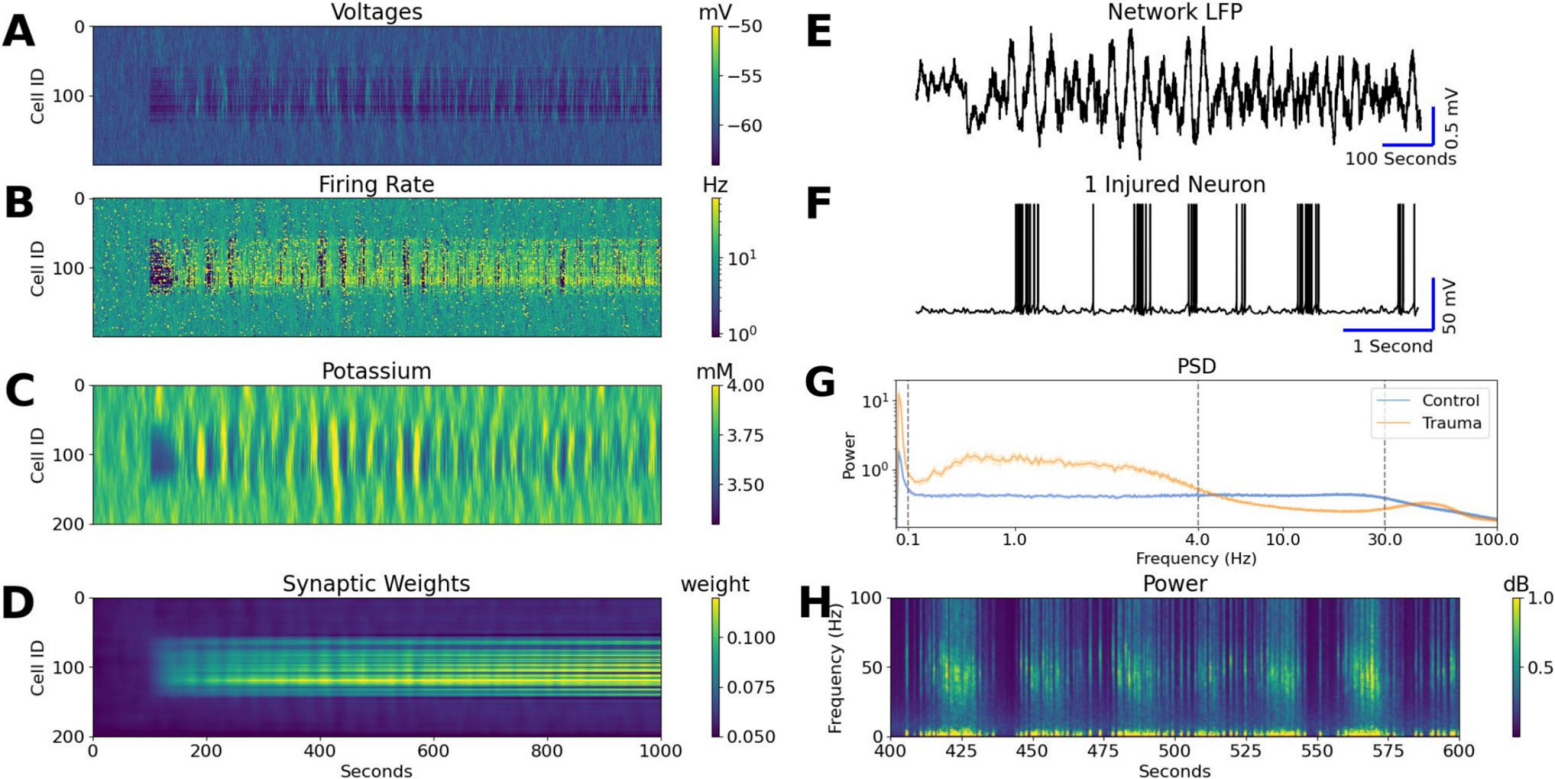
Dynamics of a network with traumatic injury inflicted at 100 seconds. (A-D) Cell voltage traces (A), firing rate (B), potassium (C), and synaptic weights (D) of a single representative model reveal dramatic changes that occur in the Injury Zone after trauma: voltages drop, firing rate increases, potassium shows large low frequency oscillations, and synaptic weights increase. (E) The network LFP shows much larger fluctuations than is seen in the control model. (F) The single neuron voltage trace shows bursting rather than tonic firing (taken from 320-325 seconds after injury has occurred). (G) A comparison of PSDs averaged over 10 trials (different random seeds) of each model (control and trauma) shows several key regions of change between control (blue) and trauma (orange): the trauma model has increased power < 0.1 Hz, < 4 Hz and 30 - 100 Hz, but decreased power 4 - 30 Hz. (H) The power spectrum over 200 seconds post injury, where the infra slow oscillations can be seen as rhythmic broadband power increases. Within these oscillations, increased Delta and Gamma power can also be seen

### Spatio-temporal dynamics in injury model

We implemented brain injury 100 seconds into a simulation as described in the Methods: a random 40 of the central-most 80 neurons had their external Poisson input reduced by 50%. This deafferented population (40 injured neurons interspersed with 40 healthy neurons; referred below as Injury Zone) in the middle of the network was bounded on either side by 60 healthy neurons (referred below as Healthy Zones). Immediately following the injury, the injured neurons temporarily became silent; the uninjured neurons in the Injury Zone revealed a drop in firing rate, but remained active (Fig. 5A). Shortly after injury had occurred, we observed an increase in amplitude of the infra slow (<0.1Hz) oscillations (Fig. 5B) and the onset of large amplitude extracellular potassium fluctuations (Fig. 5C). The primary mechanisms for these changes was HSP, i.e., the drop in the firing rate after deafferentation triggered an increase in the strength of excitatory synaptic connections to affected neurons, in an attempt to recover the target average firing rate (Fig. 5D). The slow fluctuations were most apparent in the injury zone, but also extended to the Healthy Zones. Analysis of the LFP and voltage traces of the injured cortical neurons revealed high frequency bursts of spikes (Fig. 5E/F).

We next computed the power spectrum of the membrane voltage of neurons in the Injury Zone. Compared to the power spectrum of the same neurons in the control model, we found several important changes. The first are two distinct increases in power in the infra slow (< 0.1 Hz) and Delta (1-4 Hz) ranges. We also see a decrease in power from 4 Hz to about 30 Hz, followed by another increase in the broadband Gamma power (about 30 Hz to 100 Hz) (Fig. 5G). A spectrogram over time shows infra slow oscillations in broadband power that nest high Delta and Gamma power (Fig. 5H).

**Fig. 6:**
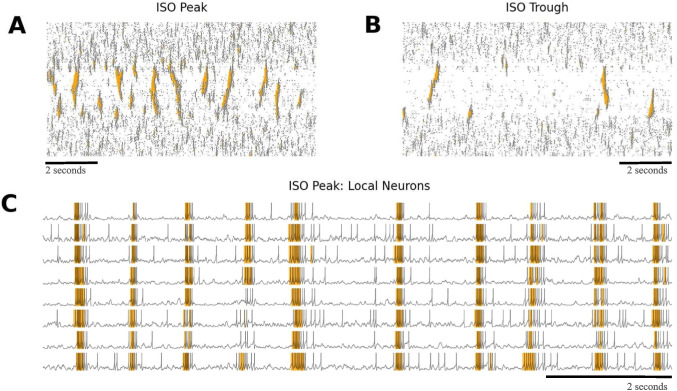
Nesting of infra-slow oscillations, Delta, and Gamma bursts. (A-B) Network activity during 10 seconds of a peak (A) or trough (B) of ISO, where Gamma bursts are plotted in orange. Gamma bursts were defined as 3 consecutive spikes with greater than 30 Hz frequency, and increase in density during the ISO peak. (C) Eight adjacent neurons’ voltage traces during an ISO peak, with Gamma bursts highlighted in orange. Bursts occur with approximately 1 Hz Delta frequency

These changes can be understood in the context of three prominent changes in the spatio-temporal network dynamics: increase in Infra Slow Oscillations (ISOs), increase in Delta power, and the emergence of 100-300 msec duration bursts of Gamma (including high-Gamma) activity, which we will refer to as Gamma bursts in the rest of the paper. The ISOs can be seen as large fluctuations in the LFP (Fig. 5E), that is mirrored in the potassium concentration (Fig. 5C). The increase in Delta power can be seen as quasi-periodic bursting in Delta frequency band (Figs. 5F, 6C). The Gamma bursts can be seen as very short bursts of high firing that synchronize across locally connected neurons (Figs. 5F, 6C). The locally synchronized bursting events sometimes spread through nearly the entirety of the Injury Zone, but did not significantly spread into the Healthy Zones (Fig. 6A-B).

ISOs, Delta power, and Gamma bursts further interact with each other; the raster plots in Fig. 6A-B taken from the peak of ISO vs the trough of a ISO show that Gamma bursts were significantly more prominent during the ISO peaks. These bursts are responsible for the increase in Gamma power, while the inter-burst timing is responsible for the increase in Delta power. The loss of power in the 4-30 Hz range comes from the loss of normal tonic firing patterns.

To further investigate the origin and characterize the infra slow and high frequency oscillations, we manually manipulated the extracellular potassium levels and synaptic weights between pyramidal neurons to determine the effects of each.

### Effect of potassium dynamics on network oscillations

**Fig. 7:**
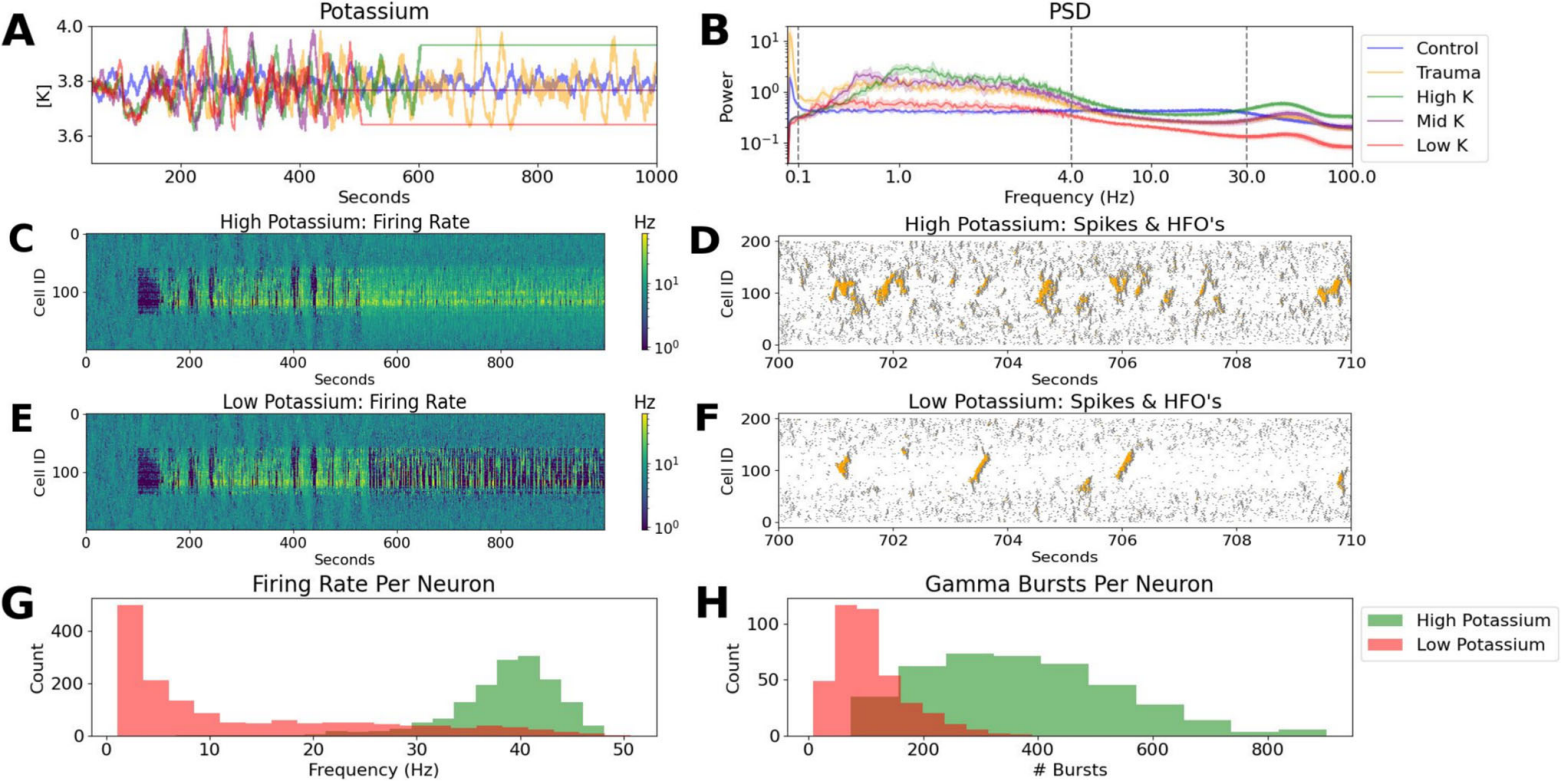
Effect of extracellular Potassium concentration on network dynamics. (A) Potassium is fixed at a high (3.93 mM), mid (3.77 mM), or low (3.64 mM) point in the native fluctuation before 550 seconds into the injured network simulation. (B) A comparison of PSDs (averaged across 10 trials) shows a broadband power increase for high potassium (green), and a broadband power decrease for low potassium (red) compared to the original trauma case (orange). (C-F) Firing rates and spike rasters with Gamma bursts highlighted in orange are shown for a representative example of a model with high potassium (C-D) and low potassium (E-F). (G-H) Distribution of firing rates and burst counts across neurons in the high/low potassium models. Neurons in the model with high levels of potassium show higher firing rates (G) and higher total number of Gamma bursts in the final 300 seconds of simulation (H) compared to neurons with low levels of potassium. All measurements take for t > 700 seconds from their respective models. Panels B, G, H are obtained by averaging across 10 trials (different random seeds)

We began by fixing the extracellular potassium level in the model at either a high (3.93 mM), middle (3.77 mM), or low (3.64 mM) point in the average potassium oscillation of the intact network (Fig. 7A). The motivation for these experiments was to identify specific contributions of the extracellular potassium dynamics to increases in oscillations in different frequency bands. Fixing the potassium concentration triggered HSP leading to a compensatory reaction in the synaptic weights. For example, if potassium was fixed at a high point, firing rate increased causing synaptic weights to decerase dramatically via HSP. Conversely, setting potassium to a low level decreased firing rates, causing synaptic weights to increase dramatically via HSP (Fig. S1). Therefore, to isolate the effects of potassium concentration dynamics, we additionally prevented the synaptic weights from undergoing further updates via HSP.

Fixing potassium at its highest point resulted in an increased neuronal firing rate across the entire network, although the highest firing remained localized to the Injured Zone (Fig. 7C). Visualization of Gamma bursts via a raster plot (Fig. 7D) showed activity very similar to that at an ISO peak in the original injured model (Fig. 6A), with a large number of bursts appearing densely throughout the Injured Zone only. As expected, fixing potassium at a low point resulted in the opposite: decreased firing throughout the network (Fig. 7E), and a lower density of Gamma bursts (Fig. 7F) akin to what was seen in an ISO trough of the original trauma model (Fig. 6B). When considering 10 simulations with different random seeds, there was a statistically significant difference between the high and low potassium models in both single neuron firing rate (t = 74.96, p < 0.001) and Gamma burst count per neuron over the final 300 seconds of the simulation (t = 30.55, p < 0.001) (Fig. 7G-H).

**Fig. 8:**
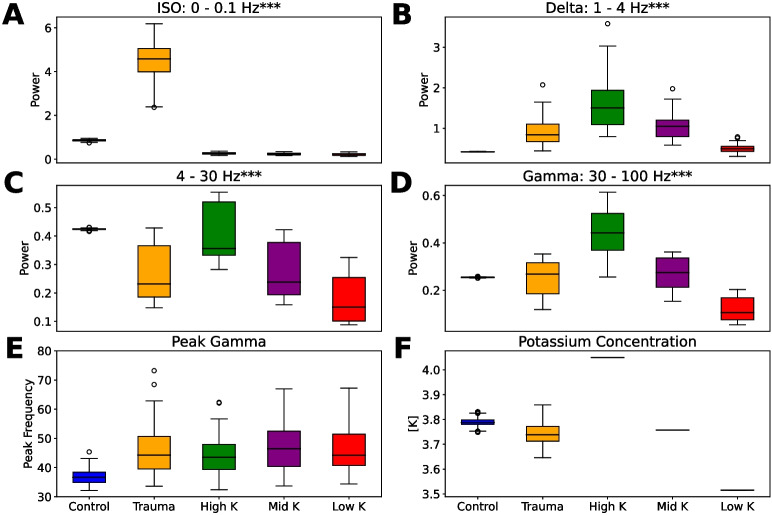
Quantification of power ($v^2 / Hz$) in several different ranges in the control (blue), trauma (orange), High K (green), Mid K (purple) and Low K (red) cases. Measurements are only taken after 650 seconds (50+ seconds after potassium levels have been fixed). HSP was blocked to prevent synaptic updates. (A) 0 - 0.1 Hz power is dramatically decreased as a result of the loss of potassium fluctuations in all fixed potassium models, but the correlation between fixed potassium levels and power is still significant (r = 0.37, p < 0.001). (B-D) 1 - 100 Hz power bands all show strong correlations between potassium level and power (B: r = 0.75 p < 0.001, C: r = 0.72, p < 0.001, D: r = 0.88, p < 0.001), showing the broad nature of Potassium related power increases. (E) There is no statistically significant correlation between potassium level and peak Gamma frequency. (F) The trauma model shows a wider distribution of potassium concentrations compared to control due to larger oscillations. All results are obtained by averaging across 10 trials (different random seeds). *** indicates a significant correlation (r > 0.3, p < 0.001) between potassium concentration and power. The box plots span from the first to third quartile of the data with a line at the median, while the whiskers extend to the most extreme non-outlier points

Analysis of the power spectrum (Fig. 7B) further revealed several important observations. In all models with fixed potassium, there was a complete loss of ISO power (<0.1 Hz) compared to the base trauma network where potassium varied freely (Fig. 8A). Note that ISO was minimal in the healthy network - therefore, a decrease in ISO power from healthy control to fixed extracellular potassium models was small (but statistically significant). Faster (>0.1Hz) oscillations were still observed in the model with fixed extracellular potassium. Specifically, from 0.1 to 100 Hz, we found strong positive correlations between fixed potassium level and band power in all ranges (Fig. 8B-D). Furthermore, despite having dramatically different density of Gamma bursts (Fig. 7H), the peak frequency in the Gamma range (30-100 Hz) did not have a significant correlation with potassium level (Fig. 8E). This implies that potassium level does not modify the intrinsic frequency of the Gamma bursts. Interestingly, control and deafferented models revealed similar average level of extracellular potassium but its fluctuations were much stronger in deafferented model (Fig. 8F).

Overall, these manipulations revealed that, in agreement with our previous study of ISO in a healthy model (Krishnan et al., 2018), it is the oscillation of extracellular potassium that controls the ISO, rather than the level of potassium. This is seen in the dramatic decrease of <0.1 Hz power after potassium is fixed at any level. We further concluded that the main effect of increasing potassium concentration is increase in power and density of Gamma bursts (Fig. 7D,H), rather than the characterization of the bursts themselves (as measured by peak Gamma frequency).

Analysis of the network with exclusively High Potassium also gives an insightful characterization of the network pathology because the two distinct states (ISO peak and ISO trough) are not being averaged together, as is the case in spectral analysis of the original trauma network. In particular, there is a much clearer increase in Gamma power over the uninjured control model (Fig. 8D) since there is no ISO trough to wash out the effects of an ISO peak.

**Fig. 9:**
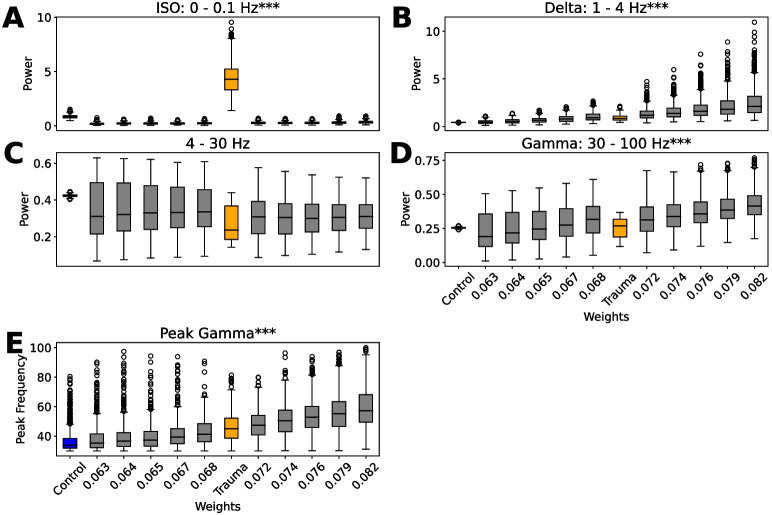
Quantification of power ($v^2 / Hz$) in several different frequency bands as synaptic weights are manually increased or decreased after injury. (A) Fixing extracellular potassium again eliminated ISO, but the correlation between fixed synaptic weight levels and ISO power remains significant (pearson’s r = 0.37 , p < 0.001). (B-D) A strong positive correlation between synaptic weights and power is seen in the Delta range (r = 0.65, p < 0.001) (B), with no correlation from 4 - 30 Hz (C), followed by another positive correlation in the Gamma range (r = 0.42, p < 0.001) (D). (E) Additionally, there is a positive correlation between the weight value and the peak Gamma frequency (r = 0.57, p < 0.001), indicating that synaptic weight level can modify the characteristics of the Gamma bursts. All results are obtained by averaging across 10 trials (different random seeds). *** indicates a significant correlation (r > 0.3, p < 0.001) between weight values and power (excluding Trauma and Control). The box plots span from the first to third quartile of the data with a line at the median, while the whiskers extend to the most extreme non-outlier points

**Fig. 10:**
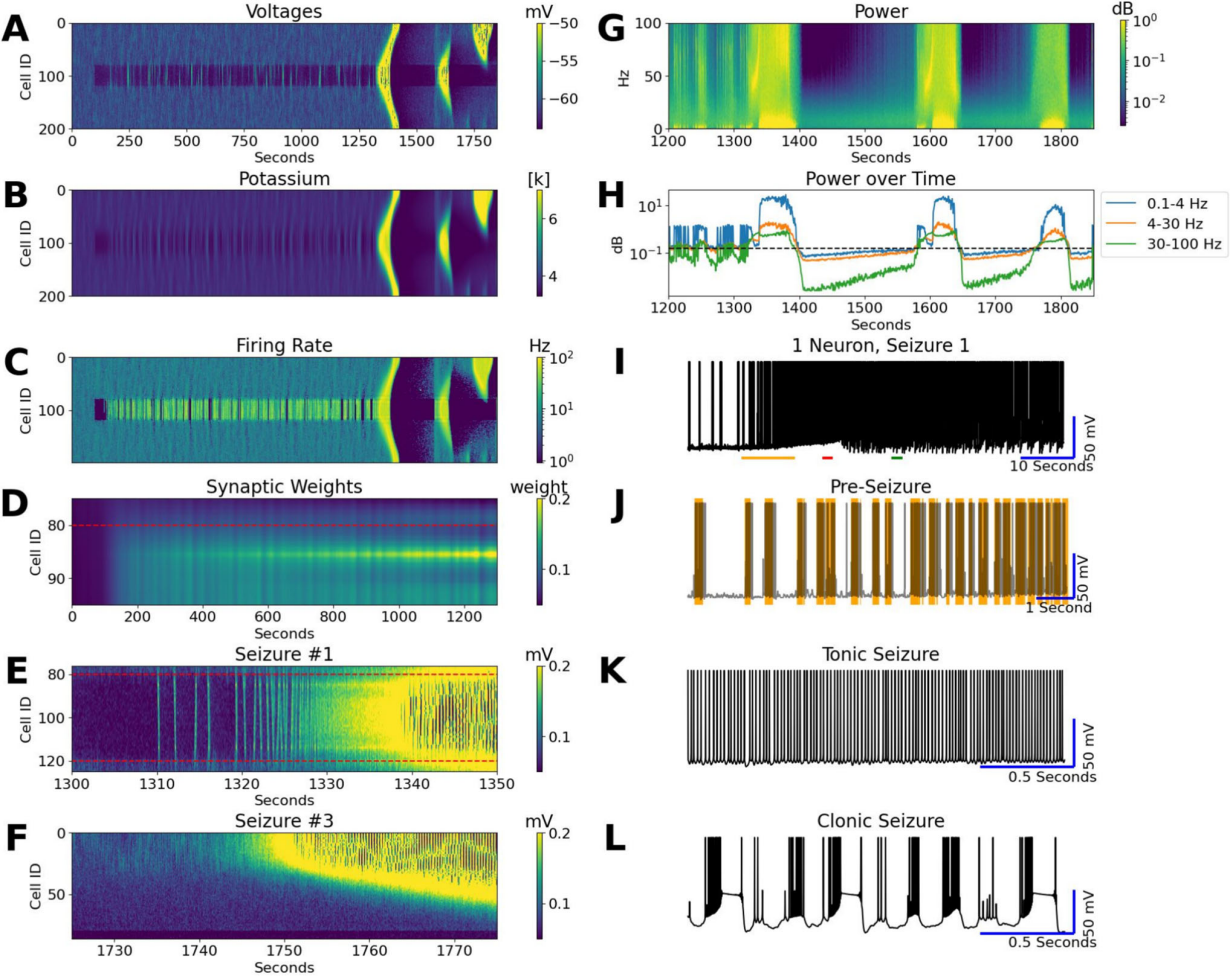
Spontaneous seizure initiation. (A) Cell voltages across the entire simulation. (B) Potassium increases strongly during seizures but decreases during postictal depression. (C) Firing Rate increases to nearly 100 Hz during seizures. D) Synaptic weights surrounding one injury border from time 0 until just before the first seizure initiates. Cells just inside the injury zone (approx. cell 85) can be seen to have the highest weight increase, while cells on either side show an initial increase followed by progressive decrease. (E-F) Further plots show cell voltages around the initiation time of first and third seizures. Red dashed lines in (D,E) indicate injury zone borders. (G) A spectrogram of the first seizure shows a dramatic increase in approx. 40 Hz Gamma band near seizure initiation. (H) Mean power in 3 frequency ranges (0.1-4 Hz, 4-30 Hz, and 30-100 Hz) over the same time period as (G). (I-L) A single cell trace of the first seizure (I) shows 3 unique stages: the buildup of Gamma bursting events before seizure initiation (orange line in the x axis, show in detail in J), tonic seizure phase (red line in the x axis, shown in detail in K), and clonic seizure phase (green line in the x axis, shown in detail in L)

### Effect of synaptic weights on gamma bursts

To delve further into the causes of Gamma bursts, we turned off HSP and varied the strength of synaptic weights up and down from the level observed after trauma (Fig. 9). This manipulation was only done on neurons in the Injury Zone. For the same reasons we fixed synaptic weights in the potassium manipulations experiments (Fig. 8), we here fixed potassium at a midpoint while the weights were manipulated, to isolate the effects of each. Synaptic weights were increased or decreased in steps of 10% of the current value (note that it gives non-linear scale on X-axis in Fig. 9).

Since potassium was fixed, we again saw a dramatic loss of <0.1 Hz power compared to the “Trauma” model, where $K^+$ was allowed to oscillate (Fig. 9A). The network oscillations in Gamma and Delta frequency bands showed moderate to strong positive correlations with synaptic weights (Fig. 9B,D), but there was no correlation between weight values and power in the 4-30 Hz range (Fig. 9C). This suggests that the strength of synaptic weights strongly and specifically affects the Gamma and Delta activities. Furthermore, there was a strong positive correlation between synaptic weights and peak Gamma frequency (Fig. 9E), indicating that weights further affect the frequency of firing within a burst, increasing the peak Gamma frequency in a way that was not seen with changes in potassium levels.

In simulations where potassium was allowed to vary freely while the weights were fixed and set at different strengths, we found that decreasing weights correlated with decreasing variability in the potassium concentration and increasing weights triggered seizure-like events throughout the network (Fig. S1). To further investigate seizure initiation and pathology, we next implemented a more severe injury that directly initiates seizures without explicit manipulations of the synaptic weights.

### Gamma bursts drive seizure initiation

To induce more severe trauma, we maintained the number of injured neurons and the percentage of input lost (50%) to each injured neuron, however, we changed the spatial pattern of the 40 injured neurons to be 100% concentrated in the center of the network rather than interspersed with healthy neurons.

After approximately 1,300 seconds of simulation time, the first spontaneous seizure-like event was initiated in the Injury Zone (Fig. 10); two more quickly followed. The spatio-temporal pattern varied between these ictal events, but all were accompanied by sustained high voltages, potassium levels, and firing rates (Fig. 10A-C). The first seizure spread throughout the whole network, while the second one remained largely local to the Injury Zone. After the second seizure, however, the ictal activity was initiated and spread entirely within the Healthy Zones (Fig. 10E-F). It suggests that once the ictal event has substantively spread outside of the Injury Zone, it can be maintained even in previously healthy parts of the network. The network potassium levels can be seen to increase dramatically during the seizure events (Fig. 10B), as reported in our previous computational and experimental studies (Frohlich et al., 2010; Krishnan et al., 2015, 2013; Filatov et al., 2011; Krishnan and Bazhenov, 2011).

Next we analyzed the initiation of ictal events. Specifically, the neurons at the edge of the injury zone (inside the injury zone, approximately 5 neurons away from the boundary) can be seen to initiate both the first and second seizure; these neurons can also be seen to have the most dramatic synaptic weight increases (Fig. 10D). Recall that each neuron has a connection radius of 5. Thus, the injured neurons at least 5 cells away from the lesion boundaries are the first within the injury region to have every cell in their connection radius also within the Injury Zone. These neurons significantly reduce activity after deafferentation and therefore experience significant HSP changes but they still receive direct input from “partially healthy”, and because of that spontaneously more active, neurons. These neurons become the seizure initiating cells. The model prediction is consistent with *in vivo* results showing high likelihood of seizure initiation near the boundary between injured and healthy cortex (Chauvette et al., 2016; Timofeev et al., 2013; Nita et al., 2007)).

A spectrogram of the first seizure shows a spike in Gamma power, followed by substantial Delta power increase (Fig. 10G), which is congruent with our previous *in vivo* observations (Grenier et al., 2003); this can be seen more clearly in the average power band traces (Fig. 10H). Analysis of the single cell voltage traces revealed that the first ictal event was preceded by increasingly frequent Gamma bursts (Fig. 10J). In subsequent ictal events, the network immediately transitioned to the next seizure after the postictal depression was recovered. Single cell voltage traces (Fig. 10I-L) further revealed the typical tonic-clonic pattern of the seizure-like event as we previously reported (González et al., 2015; Krishnan et al., 2013). During clonic phase neurons revealed transient depolarization block (Fig. 10L). This effect was observed across all neurons involved in the seizure, injured and healthy, and regardless of where the seizure initiated. Termination of the ictal event was followed by an extended period of hyperpolarization (Krishnan et al., 2013).

Why do we observe seizure-like events only in the model with a high density of injured neurons? Comparing synaptic changes in that model (Fig. 10C) with the one where injured neurons were interspersed with healthy neurons (Fig. 5D) revealed approximately 25% stronger weights in the injury zone during the same time period of 100 - 800 seconds after injury occurred. This increase in synaptic strength is statistically significant (t = -90.36, p < 0.001). This difference likely occurred because, in the model where injured neurons were interspersed with healthy neurons, the presence of healthy neurons helped maintaining a higher average firing rate, limiting HSP changes. The higher synaptic changes in the model of localized trauma led to more frequent Gamma bursts and a significant increase in extracellular potassium concentration, which was sufficient to trigger positive feedback loop mechanism leading to seizure-like events (Fröhlich et al., 2008a). Figure S2 shows average potassium fluctuations in the injury zone, along with the raster plot of the network activity preceding the first ictal event. Dense grouping of Gamma bursts around t=1250 seconds pushed potassium concentration slightly above 4 mM (which was close to the dynamic $[K^+]_o$ threshold for seizure initiation in this model (Frohlich et al., 2010)) but it was not sufficient to initiate transition. Another grouping of Gamma bursts around t=1325 sec triggered seizure initiation.

## Discussion

Traumatic brain injury (TBI) is an umbrella term that covers many different patterns and causes of injury (Saatman et al., 2008), which can affect up to 1.5 million people in the United States alone each year (Thurman et al., 1999). The link between TBI and epilepsy is well known (Chauvette et al., 2016; Avramescu and Timofeev, 2008; Dinner, 1993; Timofeev et al., 2013; Volman et al., 2011b, a), but the underlying causative network pathology is not well understood. However, infra slow oscillations (Franke et al., 2016; Guerriero et al., 2022; Huang et al., 2019), Delta power (Safar et al., 2021; Sandsmark et al., 2017; Thomasy et al., 2016; Davenport et al., 2022), and high frequency oscillations (Huang et al., 2019; Culic et al., 2005; Bailey et al., 2017; Huang et al., 2020) have all been shown to increase following TBI. In particular, high frequency power increases local to the seizure foci have been shown to precede ictal activity (Grenier et al., 2003; Arroyo and Uematsu, 1992; Worrell et al., 2004; Alarcon et al., 1995; Traub et al., 2001; Wendling et al., 2016). Our own experimental studies in cats and humans further supported the emergence of both Delta and Gamma oscillations following different types of TBI. Both experimental studies further revealed an increase of the Theta band activity. While our *in vivo* results did not show infraslow oscillations due to high pass filtering in the data processing, this has been reported previously (Franke et al., 2016; Guerriero et al., 2022; Huang et al., 2019). Our biologically realistic computational model, implementing ion concentration dynamics and homeostatic synaptic scaling, natively showed these characteristic phenomena after a simulated brain lesion, and allowed us to delve deeper into their underlying causes, interactions, and roles in seizure generation.

While the base model we used in our study was a standard Hodgkin-Huxley type network of excitatory and inhibitory neurons, two unique features were critical to model post-traumatic brain dynamics - homeostatic synaptic scaling (HSP) and ion concentration dynamics. HSP maintains brain excitability at normal levels (Volman et al., 2012). Silencing a cortical culture network for two days up-regulates synaptic excitability, while an increase in activity down-regulates excitatory synaptic efficacy (Murthy et al., 2001; Turrigiano et al., 1998; Watt et al., 2000), but not all connections (Kim and Tsien, 2008). Prolonged enhanced activity induced by the blockade of synaptic inhibition or elevated [$K^+]_{o}$ reduces the size of mEPSCs (Turrigiano et al., 1998; Leslie et al., 2001; Lissin et al., 1998). In contrast, with activity blockade, mIPSCs are scaled down, while mEPSPs are increased. After a chronic blockade of activity, intrinsic $Na^+$ currents increase and $K^+$ currents decrease in size, resulting in an enhanced responsiveness of pyramidal cells (Desai et al., 1999). Thus, HSP controls neuronal activity through the intrinsic and synaptic mechanisms (Murthy et al., 2001; Turrigiano et al., 1998). HSP also occurs *in vivo* (Desai et al., 2002). We modeled TBI as removing afferent connections to part of the network; this reduced activity and triggered HSP that modified synaptic strength. While the link between TBI and HSP was initially proposed in our earlier computational work (Fröhlich et al., 2008b; Houweling et al., 2005), this hypothesis was later confirmed *in vivo*. In cats, the connection probability was shown to increase within the undercut cortex (Avramescu and Timofeev, 2008).

Modeling the ion concentration dynamics allowed us to further link the network’s activity to its excitability. The reversal potentials of intrinsic currents are commonly assumed to be fixed in computational models. While this is an approximation even in a normal brain, the assumption becomes significantly incorrect in a pathological brain, where intense spiking or bursting can lead to considerable changes in ion concentrations, affecting both the reversal potentials and excitability. Indeed, substantial fluctuations of extracellular potassium concentration were found during electrically- or pharmacologically- induced paroxysmal activity (Heinemann and Lux, 1977). Extracellular $Ca^{2+}$ concentration ($[Ca^{2+}]_o$) reduces during epileptic seizures, thus decreasing reliability of synaptic transmission and increasing intracellular excitability (Somjen, 2002; Seigneur and Timofeev, 2011). Extracellular $Na^+$ ($[Na^+]_o$) decreases during epileptiform activity (Somjen, 2002; Meyer et al., 1961; Kraig and Nicholson, 1978; Dietzel et al., 1982), which likely corresponds to an increase of $[Na^+]_i$. Depolarizing effects of GABAa receptor activation during seizure suggest elevated levels of intracellular Cl- concentration ($[Cl^-]_i$) (Cohen et al., 2002; Timofeev et al., 2002). The changes of the ion concentrations can have profound effects on the network dynamics and are likely responsible for the characteristic patterns of electrical activity observed during seizures.

Different types and causes of injury may lead to TBI in clinical settings. Therefore, our model of simulating TBI as cortical deafferentation is certainly limited. However, it closely matches a well-established model of TBI based on cortical undercut (Timofeev et al., 2013; Nita et al., 2007; Kuśmierczak et al., 2015; Topolnik et al., 2003b; Nita et al., 2006), which we also present in our study. Furthermore, it is reasonable to assume that many different clinical models of TBI would include one or another type of synaptic injury (Jamjoom et al., 2021), which we modeled here. We used this model to investigate the underlying causes and interactions of three types of pathological network behaviors seen after TBI: (a) an increase in ISO, (b) the emergence of Gamma bursts, and (c) an increase in Delta power. We found the presence of potassium oscillations to be necessary for ISOs, but the level of potassium (low vs high) was causative only of broadband power changes. The strength of synaptic weights, however, was tied to power changes in the Delta and Gamma ranges; synaptic weight increases further increased the peak of the Gamma frequency distribution, indicating a change in the frequency of firing within the Gamma bursts. The Gamma bursts varied in spatio-temporal pattern - some remained only locally synchronized while others spread through the entire Injury Zone, but rarely entered the Healthy Zone. Importantly, Gamma bursts increased in density directly before initiation of spontaneous ictal events resembling classical spike-and-wave seizures. The later observation suggests that alternations in density of bursting events can possibly be used to predict the onset of the spontaneous seizures.

In the concentrated injury model that natively generated seizures, we found, consistent with *in vivo* results (Chauvette et al., 2016; Timofeev et al., 2013; Nita et al., 2007), the border regions of the injury to be most prone to seizure initiation - specifically, the first cells within the injury zone to be fully connected to only other injured neurons. These cells show the most dramatic increases in synaptic weights due to HSP promoting initiation of seizures. After the first seizure had occurred, however, we found that the pathological processes can spread and seizures initiate even in previously healthy parts of the network.

Our study further predicts that the frequency of Gamma bursts occurrence (but not the intrinsic frequency of these events) depends on the extracellular potassium concentration. This can be tested in slices, where extracellular potassium concentration can be controlled. Further, we predict that a buildup of Gamma bursts in cells near the injury border may be responsible for seizure initiation; this could be tested with high density electrode arrays placed near the injury zone. Indeed, experiments with cortical undercut revealed that epileptic seizures are commonly initiated near the boundary between intact and undercut brain regions (Nita et al., 2007).

The role of HSP and ion concentration dynamics in epileptogenesis and spatio-temporal patterns of epileptic seizures has been described in our previous works. Specifically, we have identified an increase in intracellular sodium and extracellular potassium levels preceding ictal events, with a marked drop in potassium during post-ictal depression (Bazhenov et al., 2004; Krishnan et al., 2015; Krishnan and Bazhenov, 2011; González et al., 2018). The sodium-potassium pump was shown to be crucial to maintaining stable dynamics (Krishnan et al., 2015), while an increase in extracellular potassium in only part of a network was found to be sufficient to generate oscillations throughout the network (Bazhenov et al., 2004). Further studies of the role of HSP have shown that sufficient synaptic up-regulation can lead to periodic slow oscillations as well as bursting activity in individual neurons (Fröhlich et al., 2008b; González et al., 2019; Krishnan et al., 2013). We have then predicted that age-related changes in the mechanism of HSP can explain differences in seizure susceptibility in older populations (Timofeev et al., 2013; González et al., 2019), and that spatially compact trauma is more prone to epileptic activity than diffuse trauma due to differential HSP mechanisms (Volman et al., 2011b, a). The novelty of our new study here is linking the mechanisms of HSP and ion concentration dynamics to an increase in Delta power and Gamma bursts after TBI but before the network alternations are sufficient to generate ictal epileptic events. Thus, the proposed model sheds light on the mechanisms of pathological brain dynamics during epileptogenesis following TBI, which could have significant clinical implications.

Model limitations include simplified neuronal morphology and the relatively small size of the network model. Furthermore, as with any computational model of the brain, it lacks some biophysical mechanisms that may influence the progression of TBI. For example, increasing evidence shows that tau aggregation, in the form of neurofibrillary tangles, is observed at autopsy following TBI. Similarly, neuroinflammation mediated by microglia is known to contribute to the development of neurodegeneration in TBI (Marklund et al., 2021; Puangmalai et al., 2024; Edwards et al., 2020). These important mechanisms may need to be taken into account in future TBI models.

Our model does not stand in isolation. Previous studies predicted the existence of both normal and abnormal high frequency oscillations (HFOs), generated by different mechanisms (Fink et al., 2015; Molaee-Ardekani et al., 2010; Traub and Bibbig, 2000; Traub et al., 2002, 2010). Importantly, it has been shown that HFOs can be generated by a variety of different network structures (Fink et al., 2015), just as we report here, observing similar pathological changes in brain electrical activity in two very different *in vivo* models of TBI. This lends support to the idea of generalizable effects of TBI despite potentially dramatically different causes and patterns of injury. Another study further identifies “resonance” modes as necessary to generate high frequency outputs; these modes are at the border of stable (normal firing) and unstable (ictal firing) regions (Molaee-Ardekani et al., 2010). This supports the idea that HFOs may play role in transitioning between healthy and pathological behavior. Finally, several models explore the cellular mechanisms of HFOs, pointing to the coupling of pyramidal neurons via gap junctions (Traub and Bibbig, 2000; Traub et al., 2002, 2010). As a whole, our own conclusions in this study both support and are supported by these results.

The effects observed natively in our model following injury closely resemble those seen in experimental studies. Given the complexity of ion concentration dynamics in our model, which are directly modeled after *in vivo* processes, it stands to reason that our simulations are reliable and relevant to true biological physiology. Our study represents another step toward a deeper understanding of the effects of TBI and how they may lead to epileptic activity. Furthermore, this model provides a testing ground for potential interventions following TBI, addressing the underlying pathology in the long term.

## Methods

### Experimental data acquisition

#### Animals

Experiments in cats were carried out in accordance with the guidelines of the Canadian Council on Animal Care and were approved by the Committee for Animal Care of Université Laval (Comité de protection des animaux de l’Université Laval, CPAUL). Surgical procedures for the sterile implantation of electrodes in cats were carried out according to methods described previously (Chauvette et al., 2011; Timofeev et al., 2001). Briefly, adult cats (3-4 years old) were pre-anesthetized with an intramuscular injection of ketamine (3 mg/kg), Buprenorphine (0.02 mg/kg), and Dexmedetomidine (5 $\mu$g/kg). The incision site was shaved, and the cats were intubated for gaseous anesthesia. Lidocaine (0.5%) and bupivacaine (0.25%) was injected at the site of the incision and in all pressure points where the head contacted the stereotaxic frame. The incision site was washed by three alternating passages of alcohol (70%) and chlorhexidine (0.5%) and the incision was performed using a scalpel. Small holes for anchoring screws (stainless steel) were drilled in the skull on each side of the head and small craniotomies were performed above both suprasylvian gyri for LFP electrode (custom-made, stainless-steel wires, 125 um diameter, Perfluoroalkoxy (PFA)-insulated) implantation. A cortical undercut was performed by entering a custom-made knife in the posterior part of the left suprasylvian gyrus through the white matter as described in detail in (Chauvette et al., 2016). The undercut size was 13-15 mm long in the posteroanterior direction and 3-4 mm wide (medioladeral axis). Two LFP electrodes were implanted in the left suprasylvian undercut gyrus, with the posterior electrode being in a very deafferented cortex and the anterior electrode being in a less deafferented cortex. An LFP electrode was implanted in the control (right) suprasylvian gyrus and an electromyogram (EMG) electrode (stainless steel wires, 125 um diameter, Perfluoroalkoxy (PFA)-insulated) was implanted in the neck muscle. All electrodes were connected to a nano-omnetic connector (Omnetics Connector Corporation, Minneapolis, MN) to which a wireless transmitter (Triangle BioSystems International (TBSI), now discontinued). All electrodes, wires and connectors were fixed in acrylic dental cement (Dentsply Canada). All electrophysiological signals were acquired on a Powerlab 16/35 (ADInstruments, Colorado Springs, CO) at 1 KHz and analyzed offline using a custom written routine in Igor Pro V6.3 (Wavemetrics, Portland , OR). The power spectral analysis was performed on 10 minutes long segments of quiet wakefulness on simultaneously recorded channels within the undercut as well as from a control area (contralateral hemisphere). Recordings used for the analysis shown in Fig. 1 were acquired 3 weeks after the undercut.

#### Humans

Additional experiments were performed in humans. Resting-state MEG (rsMEG) data acquisition was measured in alert condition using our Elekta-Neuromag VectorView MEG system with 306 MEG channels at the UCSD MEG Center. The entire recording had two 5-minute blocks with eyes closed while staying alert (Huang et al., 2020, 2014b). Data were sampled at 1000 Hz and are run through a high-pass filter [0.1 - 330] Hz cut-off. Eye blinks/movements, and heart signals are monitored.

All study participants were males and U.S. active-duty military service members or Operation Enduring Freedom / Operation Iraqi Freedom Veterans. Twenty-six participants (age 28.2 ± 6.5) had a chronic combat-related blast TBI and with persistent post-concussive symptoms for an average of 16.2 months post injury (standard deviation = 15.3; range = 4 to 84 months). Combat-related TBI was corroborated from medical records. Age-matched healthy controls included 19 individuals with similar combat experience (age 28.5 ± 5.9), but without a significant history of concussion based on self-report.

All TBI participants were evaluated in a clinical interview to assess the nature of their injuries. The diagnosis of TBI was based in part on standard Veterans Affairs and Department of Defense diagnostic criteria (Management of Concussion/mTBI Working Group, 2009): 1) loss of consciousness < 30 minutes or transient confusion, disorientation, or impaired consciousness immediately after the combat-related trauma; 2) post-traumatic amnesia < 24 hours; and 3) an initial Glasgow Coma Scale (Teasdale and Jennett, 1974) between 13-15 if available. Since the Glasgow Coma assessment was not accessible in combat theater, participants missing an assessment, but who met other inclusion criteria, were also enrolled.

Conventional structural MRI acquisition and the construction of the BEM: Using a 3T GE Discovery MR750 MRI scanner, we acquired a high-resolution MRI volume with a resolution of 1$\times$1$\times$1 mm$^{3}$ using a T1-weighted 3D-IRSPGR pulse sequence. Gradient nonlinearity spatial distortion in MRI was corrected (Jovicich et al., 2006). The T1-weighted images were used to construct a source grid for Fast-VESTAL and a boundary element method (BEM) model for MEG forward calculation (Mosher et al., 1999). Conventional MRI sequences for identifying TBI lesions in participants were also performed: 1) Oblique T2*-weighted; 2) Oblique T2-weighted with ASSET; 3) Oblique FLAIR; 4) Oblique DWI. Susceptibility-weighted imaging was also performed to detect subtle blood products.

The voxel-wise MEG source magnitude images were obtained using our high-resolution Fast-VESTAL MEG source imaging method following the previously described procedure (Huang et al., 2020, 2014a). In each participant: the data in each epoch were first DC-corrected and then run through band-pass filters for the delta band (1 - 4 Hz). In all participants, voxel-wise whole brain MEG source magnitude images obtained from Fast-VESTAL were first spatially co-registered to the MNI-152 (Grabner et al., 2006) brain-atlas template using a linear affine transformation program, FLIRT, from FSL software (www.fmrib.ox.ac.uk/fsl/) (Smith et al., 2004; Woolrich et al., 2009). Once in MNI-152 space, the MEG source magnitude images were spatially smoothed using a Gaussian kernel with 5 mm full width half maximum, followed by a logarithmic transformation using FSL.

A voxel-wise, two-tailed ANOVA was then performed to test for differences between the mTBI and control groups. Family-wise errors across voxels were controlled by using a standard cluster analysis for the ANOVA F-value maps. In this approach, the uncorrected threshold for voxel-wise t-test maps was set at p < 0.01, and the cluster size was determined by “3dFWHMx” and “3dClustSim” functions from AFNI software (http://afni.nimh.nih.gov). A mask that contained the statistically significant clusters was created, and then applied to the ANOVA maps to create the corrected group statistical maps (p < 0.01) for the MEG source magnitude images. The above procedure is similar to the one described in Huang et al. (2020) , except here ANOVA (instead of t-test) and delta-band activity (instead of gamma-band activity) were used.

### Model design

Our computational model consists of 200 pyramidal neurons and 40 inhibitory neurons. This model is directly an expansion of the model presented in (González et al., 2015); in addition to a larger number of neurons being modeled, HSP is implemented as a local (rather than global) process so that localized effects of injury can be examined. The simulated neurons are conductance-based two compartmental neuron models with realistic ion concentration dynamics, as described in many previous publications (Fröhlich et al., 2008b; González et al., 2019, 2015; Houweling et al., 2005; Volman et al., 2011b, a; Bazhenov et al., 2008, 2004, 2002; Frohlich et al., 2010; Fröhlich et al., 2006; Fröhlich and Bazhenov, 2006; Krishnan et al., 2018; Krishnan and Bazhenov, 2011; González et al., 2018; Krishnan et al., 2015). The membrane potential dynamics for each compartment were modeled by the following equations:

1 $$\begin{aligned} C_m \frac{dV_D}{dt} = -g_c^D(V_D - V_S) - I_D^{leak} - I_D^{pump} - I_D^{int} \end{aligned}$$

2 $$\begin{aligned} g_c^S(V_D - V_S) = - I_S^{leak} - I_S^{pump} - I_S^{int} \end{aligned}$$

where $V_D$, $V_S$ are the dendritic and axosomatic membrane potentials, $I_D^{leak}$, $I_S^{leak}$ are the sum of the ionic leak currents, $I_D^{pump}$, $I_S^{pump}$ are the sum of the $Na^+$ and $K^+$ currents, and $I_D^{int}$, $I_S^{int}$ are the intrinsic currents for the dendritic and axosomatic compartments. Reversal potentials for all currents are calculated using Nernst equation based on the intra- and extracellular ion concentrations. The intrinsic currents for the dendritic and axosomatic compartments as well as ion concentration dynamics for $[K^+]_o$, $[K^+]_i$, $[Na^+]_o$, $[Na^+]_i$, $[Ca^{2+}]_i$, and $[Cl^-]_i$ have been previously described (González et al., 2015; Krishnan and Bazhenov, 2011; Krishnan et al., 2015). Briefly, the ion concentrations were determined by leak currents, intrinsic currents, pump-mediated currents, extracellular diffusion, and glial activity. We here focus particularly on potassium - the evolution of $[K^+]_o$ is given by the following equations:

3 $$\begin{aligned} \frac{d[K^+]_o}{dt} =&- (\frac{\kappa }{F})(I^{Int}_{\sum K} + I_\kappa ^{pump}) + G \end{aligned}$$

4 $$\begin{aligned}&+ \delta _o (\frac{[K^+]_{o-1} + [K^+]_{o+1}}{2} \nonumber \\&- [K^+]_o) + \delta _0 ([K^+]_{oc} - [K^+]_o) \nonumber \\ G =&\kappa _1 \frac{[B]_{max} - [B]}{\kappa _{1N}} - \kappa _2 [K^+]_o[B] \end{aligned}$$

5 $$\begin{aligned} \frac{d[B]}{dt}=&\kappa _1 ([B]_{max} - [B]) - \kappa _2[K^+]_o[B] \end{aligned}$$

where $\kappa$ (conversion factor) = 10, F = 96489 C/mol, d = 0.15, determined the ratio of the volume of the extracellular compartment to the surface area, $[K^+]_{oc}$ is the concentration of $K^+$ in the adjacent compartment, $[K^+]_{o-1}$ and $[K^+]_{o+1}$ are the concentration from neighboring cells. Glial $K^+$ uptake current was modeled by a free buffer (total buffer $[B]_{max}$ = 500 mm) with concentration [B], which bound and unbound from $K^+$ based on the first-order kinetics with rates $\kappa 1$ and $\kappa 2$ given by $\kappa 1 = 0.008$ and $\kappa 2 = \kappa 1 / (1 + \exp (\frac{[K^+]_o - [K^+]_{oth}}{-1.15}))$

The equations for the other three concentrations are given as follows:

6 $$\begin{aligned} \frac{d[K^+]_i}{dt} =&- (\kappa / F)(I_{\sum K}^{Int}+ I_K^{pump}) + \delta _i([K^+]_{ic} - [K^+]_o) \end{aligned}$$

7 $$\begin{aligned} \frac{d[Na^+]_o}{dt} =&(\kappa / F_D)(I_{\sum Na}^{Int}+ I_{Na}^{pump}) + \delta _o([Na^+]_{o-1} - [Na^+]_{o+1}) / 2 \end{aligned}$$

8 $$\begin{aligned}&- [Na^+]_o - \delta _0([Na^+]_{oc} - [Na^+]_o) \nonumber \\ \frac{d[Na^+]_i}{dt} =&-(\kappa / F)(I_{\sum Na}^{Int}+ I_{Na}^{pump}) + \delta _i([Na^+]_{oc} - [Na^+]_o) \end{aligned}$$

where $[Na^+]_{oc}$ is the concentration of $Na^+$ in the adjacent compartment, $[Na^+]_{o-1}$ and $[Na^+]_{o+1}$ are the concentration from neighboring cells.

The 200 pyramidal and 40 inhibitory neurons are arranged linearly. Each pyramidal cell has a connection radius of 5 to other pyramidal cells (with AMPA conductance strength of 3.5 nS and NMDA conductance of 0.9 nS) and 1 inhibitory cell (with AMPA and NMDA conductance strengths of 2.4 nS and 0.24 nS), while inhibitory cells make a total of 5 unique connections to pyramidal cells (with GABAA conductance strengths of 3.5 nS). This means each pyramidal cell receives input from 10 other pyramidal cells and 1 inhibitory cell. Each neuron additionally receives external Poisson input as baseline activity to simulate long range connections from other cortical regions as described in our previous studies (González et al., 2015; Krishnan and Bazhenov, 2011; Krishnan et al., 2015).

Homeostatic synaptic plasticity (HSP) is implemented to maintain a firing rate of 5 Hz by adjusting AMPA conductances between excitatory neurons. The relevant firing rate for each neuron is calculated as the mean firing rate of itself and all pyramidal cells within its connection radius. AMPA conductances are then scaled relative to the difference between the current ($\bar{v}$) and target ($v_0$) firing rates (Eq. 9). The HSP scaling factor $\alpha _{HSP}$ is here set to 0.01, and *k* represents subsequent timesteps.

9 $$\begin{aligned} AMPA_{k+1}^{PY-PY} = AMPA_{k}^{PY-PY} - \alpha _{HSP} * (v_0 - \bar{v}) * AMPA_{k}^{PY-PY} \end{aligned}$$

Traumatic Brain Injury is modeled as a reduction to a cell’s Poisson input, to represent lost synaptic connections and broken axons resulting from injury. We here primarily implemented injury as a 50% loss of Poisson input. Injury was applied to a random 40 of the central 80 neurons in the model; these central 80 neurons will be referred to as the Injury Zone. This means that there are 60 healthy neurons on either side of the Injury Zone; these regions will be referred to as the Healthy Zones. We further implemented a more concentrated injury that leads to seizures: in this case, injury was applied to all of the most central 40 neurons, with no healthy neurons interspersed.

This paper identifies and characterizes three types of pathology seen after injury: Infra Slow Oscillations (ISOs), Gamma bursts, and increased Delta power. ISO’s are here defined as <0.1 Hz oscillations. Gamma bursts are here defined as sequences of spikes that have an interspike interval less than 33 ms (greater than 30 Hz) for a minimum of 3 consecutive spikes. This was done to exclude single random high frequency spikes. These bursts often contain much higher frequencies (up to 80 Hz) but this method was chosen to identify as many events as possible while excluding any behavior seen in the control model or pre-injury timeframes.

Power spectrums of the network are computed using Welch’s method (Solomon, 1991) with a sliding window of 50 seconds with 50% overlap in order to capture low frequency events. The power spectrum is computed (in units of $v^2 / Hz$) for groups of 5 linearly consecutive neurons, then averaged over groups; this ensures retention of high frequency events, while also getting a spatial average. The local field potential is computed as the smoothed average of all pyramidal neuron voltages.

### Data analysis

We first characterized basic features of a control network and an injured network - cell voltage traces, firing rates, and power spectrums. Then, to investigate the effects of potassium and synaptic weights, we applied several different types of manipulations. First, potassium and synaptic weights were fixed, or prevented from further changes at a given point after injury had occurred and pathological behavior had been established. Then, one of the two (potassium or weights) could be set to different levels to investigate the effects of such changes. It is important to note that these experiments require both potassium and the synaptic weights to be fixed before one can be manipulated or set to an extreme; if one is left to vary freely while the other is manipulated, the first will show large compensatory effects. For example, when potassium is set to a high level, the weights decrease correspondingly (Fig. S1). It is then impossible to say if the observed changes are due to high potassium or low synaptic weights. With this procedure in place, we can then determine the role of each in the observed changes in network behavior after injury.

We ran statistical analysis on the observed changes in power across different levels in potassium and synaptic weights, using 10 stochastic replications of each model. This was done using Pearson’s R to determine the relationship between potassium (or synaptic weight) values and power in several different frequency bands, with significance thresholds of r > 0.3 and p < 0.001. Furthermore, an independent t-test was used to determine statistically significant changes in 1) firing rate and Gamma burst density between High and Low potassium models, and 2) weight increases in the diffuse v.s. concentrated injury models.

## Supplementary Information

Below is the link to the electronic supplementary material.

Supplementary file 1 (pdf 296 KB)

## Data Availability

The data from the computational models described in this paper can be accessed at https://gin.g-node.org/bmduffy/TBI.
