# Supplementary material for: Network effects of traumatic brain injury: from infra slow to high frequency oscillations and seizures
Source: J Comput Neurosci. 2025 Feb 28;53(2):247–66. doi: 10.1007/s10827-025-00895-5 (PMC12181067; doi:10.1007/s10827-025-00895-5)
Supplement: Supplementary file 1 — (pdf 296 KB) [file 10827_2025_895_MOESM1_ESM.pdf]

<u>Network effects of traumatic brain injury: from infra slow to high frequency oscillations</u>

Journal of Computational Neuroscience

Brianna Marsh, Sylvain Chauvette, Mingxiong Huang, Igor Timofeev, Maxim Bazhenov*

*mbazhenov@health.ucsd.edu, The University of California San Diego

SUPPLEMENTAL INFORMATION

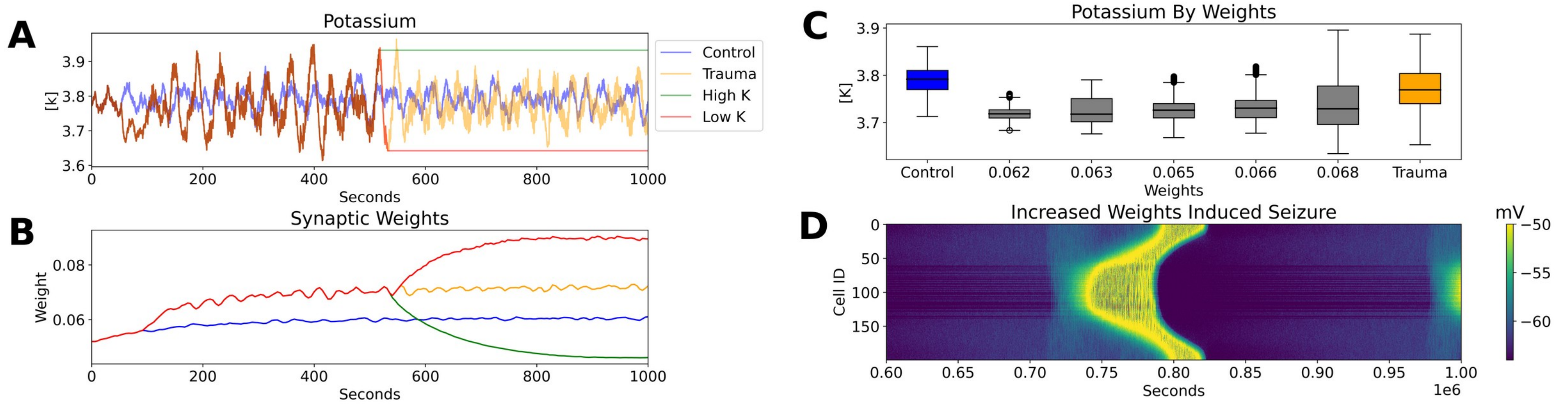

Figure S1: Compensatory Reactions of Potassium and Synaptic Weights. When potassium is frozen at an extreme value (A), there is a strong compensatory reaction in synaptic weights (B). (C) Potassium levels and variability by fixed synaptic weight value – with weights above default Trauma levels, networks go into seizure-like states as shown in (D) - network voltages when synaptic weights are increased by 10% from Trauma

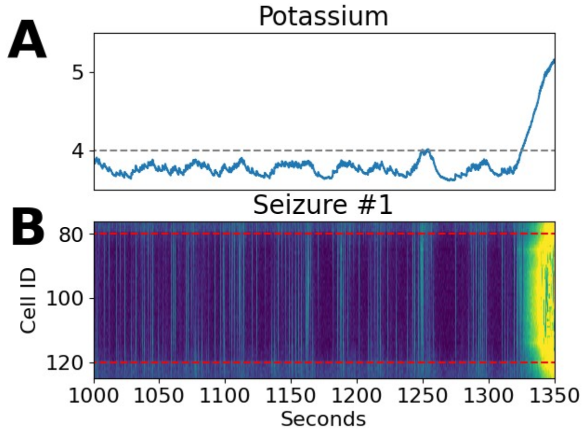

Figure S2: Seizure Initiates Above Potassium Threshold. (A) Network average potassium oscillations and (B) cell voltages preceding the first seizure at approximately 1325 seconds. The potassium fluctuation can be observed to remain largely below 4 mM during interictal activity. Transient increases in extracellular potassium created conditions for seizure initiation. At around 1325 seconds, the K^+ increase was significant enough to trigger a positive feedback loop between neuronal firing and K^+-dependent excitability, leading to seizure initiation.
